# Relational coordination in value-based health care

**DOI:** 10.1097/HMR.0000000000000381

**Published:** 2023-08-19

**Authors:** Dorine J. van Staalduinen, Petra E.A. van den Bekerom, Sandra M. Groeneveld, Anne M. Stiggelbout, M. Elske van den Akker-van Marle

**Affiliations:** Dorine J. van Staalduinen is MSc, PhD Candidate, Department of Biomedical Data Sciences, Leiden University Medical Center, and Institute of Public Administration, Leiden University, the Hague, the Netherlands. E-mail: d.j.van_staalduinen@lumc.nl.; Petra E.A. van den Bekerom, Assistant Professor, Institute of Public Administration, Leiden University, the Hague, the Netherlands.; Sandra M. Groeneveld is Professor, Institute of Public Administration, Leiden University, the Hague, the Netherlands.; Anne M. Stiggelbout is Professor, Department of Biomedical Data Sciences, Leiden University Medical Center, the Netherlands, and Erasmus School of Health Policy & Management, Erasmus University Rotterdam, the Netherlands.; M. Elske van den Akker-van Marle is Assistant Professor, Department of Biomedical Data Sciences, Leiden University Medical Center, the Netherlands.

**Keywords:** Boundary spanning behavior, integrated practice unit, interprofessional collaboration, relational coordination, team meetings, value-based health care

## Abstract

**Background:**

An important element of value-based health care (VBHC) is interprofessional collaboration in integrated practice units (IPUs) for the delivery of the complete cycle of care. High levels of interprofessional collaboration between clinical and nonclinical staff in IPUs are assumed rather than proven. Factors that may stimulate interprofessional collaboration in the context of VBHC are underresearched.

**Purpose:**

The aim of this study was to examine relational coordination (RC) in VBHC and its antecedents.

**Approach:**

A questionnaire was used to examine the association of both team practices and organizational conditions with interprofessional collaboration in IPUs. Gittell’s Relational Coordination Survey was drawn upon to measure interprofessional collaboration by capturing the relational dynamics in coordinated working. The questionnaire also included measures of team practices (team meetings and boundary spanning behavior) and organizational conditions (task interdependence and time constraints).

**Results:**

The number of different professional groups participating in team meetings is positively associated with RC in IPUs. Boundary spanning behavior, task interdependence, and time constraints are not associated with RC.

**Conclusions:**

In IPUs, the diversity within interprofessional team meetings is important for establishing high-quality communication and relationships.

**Practice Implications:**

Hospital managers should prioritize facilitating and encouraging shared meetings to enhance RC levels among professional groups in IPUs.

Value-based health care (VBHC) is a management strategy that is receiving growing attention in health care organizations (Nilsson et al., 2017). The argument for shifting from volume-based health care to VBHC practices was introduced by Porter and Teisberg (2006). According to their reasoning, VBHC achieves the best outcomes for the patient at the lowest cost. An important element of the VBHC strategy is organizing care in integrated practice units (IPUs): “In an IPU, a dedicated team made up of both clinical and nonclinical personnel provides the full cycle of care for the patient’s condition” (Porter & Lee, 2013, p. 5). An example could be a breast cancer IPU. Clinicians with different medical expertise (e.g., surgeons and oncologists), supportive care professionals (physiotherapists and dieticians), and other functionaries (secretaries) are needed that all manage a certain aspect of the full cycle of breast cancer care. All involved employees are considered members of the breast cancer IPU.

According to the VBHC literature, interaction between the various clinical and nonclinical staff in an IPU is considered vital for successful VBHC delivery (Porter & Lee, 2013). Research points the importance of coordination and communication in the functioning of health care organizations, especially for maintaining effective interprofessional collaborations (Nilsson et al., 2017; Zwarenstein et al., 2009). In the context of an IPU, interprofessional cooperation between hospital departments is particularly crucial (Nilsson et al., 2017). However, research on managing and stimulating such collaboration in VBHC scenarios is lacking (van Staalduinen et al., 2022) and especially so on the coordination of tasks within IPUs (Fredriksson et al., 2015; Nilsson et al., 2017). Furthermore, factors that influence collaboration between IPU members from different professional backgrounds are insufficiently researched. Identifying these factors is essential for both theory and practice to improve interprofessional collaboration in IPUs. This study aims to fill this gap by identifying and describing factors that are associated with interprofessional collaboration in IPUs.

In examining interprofessional collaboration in IPUs, we draw on relational coordination (RC) theory (Gittell, 2002b, 2006). RC relates to optimal communication between participants in delivering high-quality care and emphasizes the importance of relationships across professional boundaries (Gittell et al., 2013). RC is defined as a “mutually reinforcing process of interaction between communication and relationships carried out for the purpose of task integration” (Gittell, 2002a). The quality of communication, together with the quality of relationships, constitutes the level of RC among team members. Given that RC has been shown to enhance the effectiveness of interprofessional collaboration (Bolton et al., 2021), it is important to understand how RC can be improved. Consequently, this article focuses on antecedents of RC in IPUs, specifically those that are subject to managerial scrutiny.

## Theory

### Interprofessional collaboration in the context of VBHC

Reorganizing the way professionals deliver care is at the core of a shift from volume-based health care to VBHC (Porter & Lee, 2013). This requires the change from care delivered in silos organized around medical specialties to care organized around the patient’s medical condition. IPUs should be interprofessional collaborations consisting of care providers from different clinical and nonclinical professional backgrounds, who treat patients over the full cycle of care (Morrice et al., 2020; Porter & Lee, 2013). Interprofessional collaboration involves health care workers from two or more professions and wherein medical specialists with divergent expertise are classified as part of the same profession (Prentice et al., 2015). VBHC theory emphasizes that an IPU should consist of experts who know and trust each other, meet frequently, and review data on their IPU’s performance (Porter & Lee, 2013).

### Relational coordination

RC can be seen as a way to understand the relational dynamics in coordinating work within interprofessional collaborations (Gittell, 2002b). RC addresses two important aspects of the work of professionals working in IPUs: communication and relationships (Gittell, 2006). A team that collaborates effectively has frequent, accurate, timely, and problem-solving communication and relationships based on shared goals, shared knowledge, and mutual respect (Gittell, 2006). High-quality communication has been shown to be crucial for accomplishing shared goals, sharing knowledge and achieving mutual respect within a collaboration (Gittell, 2006). In turn, having relationships that involve shared goals, shared knowledge, and mutual respect positively influences the quality of communication. In contrast, infrequent, delayed communication in which there is a culture of finger pointing creates relationships where the goals are not shared, knowledge is not spread, and disrespect is present (Gittell, 2006; Gittell et al., 2020). As such, in RC theory, aspects of communication and relationships are mutually reinforcing.

### Team practices and RC

Gittell’s conceptual model proposes several relational work practices that influence RC. We have taken two team-level practices (team meetings and boundary spanning behavior) from that model (Bolton et al., 2021; Gittell, 2002a) and tested their association with RC in IPUs. We selected these two work practices based on our argument that health care managers can exert influence over them.

Team meetings give employees the opportunity to directly exchange tasks and ideas (Gittell, 2002b; Schölmerich et al., 2014). The meetings will facilitate interaction between the different professional groups that are represented within a team, thereby increasing the opportunity to communicate and coordinate (Galbraith, 1974). Furthermore, when team members from different professional groups are present at these team meetings, they can build a shared knowledge base, receive accurate information, and build strong relationships (Schölmerich et al., 2014). Research on health care teams has already shown that having more professional groups participating in interprofessional team meetings contributes to increasing the level of RC (Hartgerink et al., 2014). Based on the above, we hypothesized:

*H1: The number of different professional groups in team meetings is positively associated with RC in IPUs.*

Those individuals who engage in boundary spanning behavior “coordinate interdependent work efforts and bridge disconnected parties by actively managing relationships external to the team itself” (Marrone, 2010). Furthermore, team members’ boundary spanning behaviors tend to link groups of professionals across boundaries, transfer relevant information across professional groups, and improve coordination between professional groups that are divided by location or function (Van Meerkerk & Edelenbos, 2014).

An IPU requires the coherent coordination between its team and its external environmental requirements. Whereas the members of the IPU are the internal environment, the external environment comprises the various departments to which the IPU members still belong. Coordination and information exchange are necessary between demands within the IPU (internal) and needs of departments outside the IPU (external). This cross-boundary integration of work practices requires active participation of IPU members who engage in boundary spanning activities. This boundary spanning behavior of IPU members may therefore reduce inefficiencies, increase coordination, and facilitate interaction between professional groups (Gittell, 2002a; Gittell & Weiss, 2004). Earlier research found that cross-functional health care teams that have high levels of boundary spanning behavior report higher levels of RC (Gittell, 2000, 2002b). It is therefore expected that, in IPUs where the professionals belong to different departments and require coherent coordination, boundary spanning behaviors will positively influence the quality of RC. On this basis, the following hypothesis was formulated:

*H2: Boundary spanning behavior by IPUs members is positively associated with RC in IPUs.*

### Organizational conditions and RC

#### Direct effects of organizational conditions

Gittell (2002a, 2006) argues that RC can be expected to have a greater impact on performance under conditions of task interdependence and time constraints because it is a form of coordination that supports participants in collaborating effectively. However, as argued below, these conditions might also directly influence RC.

Task interdependence refers to the extent to which team members have to interact with each other in order to complete tasks (Langfred, 2000). Health care delivery is characterized by a large degree of interdependence, where the complexity of health care practices calls for the integration of the knowledge and tasks of various professionals (Sangaleti et al., 2017), and consequently, professionals within an interprofessional team are considered dependent on the other professionals within the team to accomplish their goals. High task interdependencies demand effective communication and strong relationships (Gittell, 2006). The need for RC is thus more strongly felt where there is task interdependence, implying a direct effect of task interdependence on RC. Earlier research suggests that professionals who perceive high levels of task interdependence are more inclined to improve their communication and knowledge exchange (Johnson & Johnson, 2002). Conversely, a lack of perceived interdependence between professionals may decrease their perceived need and motivation to actively interact (Tindale, 2002). Therefore, we propose that:

*H3: In IPUs, task interdependence is positively associated with RC.*

Time constraints are often mentioned as a challenge in health care (Reeves et al., 2009). Research shows that time constraints can negatively influence interprofessional collaboration by causing communication between professionals to become terse and incoherent (Soukup et al., 2020). Furthermore, the literature emphasizes the importance of having sufficient time to build and maintain relationships between professionals (Tsasis et al., 2012). Given the abovementioned evidence, professionals’ time constraints are expected to harm communication and relationships in IPUs. We therefore hypothesize that:

*H4: In IPUs, time constraints are negatively associated with RC.*

#### Moderating effects of organizational conditions

Gittell’s (2002b) conceptual model proposes that the contingency factors of task interdependence and time constraints moderate the effect of RC on quality and performance outcomes (Gittell, 2002b). Below, we argue that these contingency factors also moderate the positive association of the number of different professional groups present at team meetings and the boundary spanning behavior with RC.

First, we expect the number of different professional groups present at team meetings to have a greater impact on RC under conditions of high task interdependence. If the tasks of professionals within a team are highly interdependent, the professionals will be dependent on each other’s specific and professional knowledge to accomplish the team’s goals. This increased dependence on other professionals will affect the importance of meaningful interprofessional collaboration (Gu et al., 2018), such as attending team meetings and engaging in boundary spanning activities. In IPUs where professionals rely on each other to achieve their goals, the coordination of tasks is expected to be more important for effective interprofessional collaboration compared to IPUs where professionals work independently. Therefore, we expect that the presence of diverse professional groups in team meetings and the occurrence of boundary spanning behavior will have a more significant impact on RC when team members are task interdependent. As such, we would expect team meetings and boundary spanning behavior to be more effective in a context where there is a high task interdependence. This leads to the following hypotheses:

*H5: High levels of task interdependence reinforce the association between the number of different professional groups in IPU team meetings and RC.*
*H6: High levels of task interdependence reinforce the association between boundary spanning behavior by IPU members and RC within the IPU.*

Second, we would expect team meetings and boundary spanning behavior to have less impact on RC under time constraints. Time constraints refer the lack of time relative to the time required to successfully complete a task. Under such conditions, one could expect IPU members to be less focused and have less time to establish the desired interactions with others from both within and beyond the IPU. Professionals experiencing time constraints are expected to communicate less accurately and be rushed (Allen & Hughes, 2017). Being rushed can lead to having less effective interactions with other professionals, such as through meaningfully engaging in team meetings and in boundary spanning behavior. For instance, although professional groups are present at team meetings and boundary spanning behavior occurs, the quality of such interactions may suffer due to time constraints. This is expected to result in a smaller effect of these team practices on RC. Therefore, we expect team meetings and boundary spanning behavior to be less effective in a context of increased time constraints. We therefore formulate the following hypotheses:

*H7: Time constraints weaken the association between the number of professional groups that are present at team meetings and RC in IPUs.*
*H8: In IPUs, time constraints weaken the association between the boundary spanning behavior of IPU members and RC.*

## Method

The setting for this cross-sectional study was in IPUs^1^ in two academic hospitals in the Netherlands. The hospitals’ independent medical ethics committees confirmed that the study did not fall within the Medical Research Act, thereby indicating that ethical approval was not needed. To be eligible to participate in the study, a respondent had to be part of an IPU. We asked eligible employees to complete an online survey regarding the interprofessional collaboration in their IPU. The survey included measures of RC, team practices (team meetings and boundary spanning behavior), and organizational conditions (task interdependence and time constraints; see Supplementary Appendix A, http://links.lww.com/HCMR/A129). Demographic information was gathered regarding respondents’ gender, age, profession, and education. Table 1 shows descriptive statistics and correlations. Eligible professionals (*N* = 235) received the online survey in January (Hospital 1) or December 2021 (Hospital 2). In total, 86 employees from 26 IPUs participated in the survey (37% response rate). The number of professionals per IPU, as reported by the hospitals, ranged from 4 to 35. Most of the respondents were medical specialists (59%, *n* = 51), followed by nurses (23%, *n* = 20), allied health professionals (11%, *n* = 6), and administrative employees (7%, *n* = 9).

**Table 1:** Descriptive statistics and correlations for all variables in the analyses (*n* = 86)

	Mean (*SD*)	Range	1	2	3	4	5	6	7	8	9
(1) Gender (male = 1)	0.63	0–1	1								
(2) Age	47.00 (10.84)	25–66	−.121	1							
(3) Education (university degree = 1)	0.65 (0.48)	0–1	−.549**	−,059	1						
(4) Profession (medical specialist = 1)	0.59 (0.49)	0–1	−.540**	.148	.861**	1					
(5) Boundary spanning behavior	3.56 (0.95)	1–5	−.026	.171	.103	.167	.1				
(6) Team meetings	3.53 (0.75)	2–5	.027	−.059	.075	.077	.138	1			
(7) Task interdependence	4.22 (0.57)	2–5	−.268*	.125	.338**	.283**	.165	.236*	1		
(8) Time constraints	3.41 (1.00)	1–5	.025	.102	.169	.125	.157	.221*	.151	1	
(9) Relational coordination	3.60 (0.66)	1.21–4.89	−.171	.187	.129	.132	.132	.293**	.172	.156	1

*Note.* Variable “profession”: the reference category 0 includes nurses, administrative employees, and allied health professionals.

*p < .05.

**p < .01.

RC was measured using the Dutch translation of Gittell’s Relational Coordination Survey (Van Houdt et al., 2009). Respondents were asked to evaluate their communication and relationships with other professional groups within their IPU by indicating their agreement with statements using a Likert scale ranging from 1 (*never*) to 5 (*always*) for each professional group (medical specialist, nurse, allied health professional, and administrative employee). An individual’s total score was calculated by taking the average of the seven RC dimensions (frequent, accurate, timely, and problem-solving communication plus shared goals, shared knowledge, and mutual respect. RC scores for each dimension (communication and relationships) were also calculated as the average of the respective items for each respondent. The Cronbach’s alpha coefficients indicated a good reliability for the overall RC scale and for its subdimensions: overall (.85), communication (.83), and relationships (.84).

The instrument used to measure team meetings was a single-item measure adapted from Deneckere et al. (2012), which aligns with Gittell’s conceptualization of team meetings (Deneckere et al., 2012; Gittell, 2002a). Respondents were asked to indicate the frequency of attendance at IPU meetings for each professional group (medical specialist, nurse, allied health professional, and administrative employee) using a 5-point Likert scale ranging from *never* (1) to *always* (5). The variable used in the analyses represents the number of professional groups that *always* attend team meetings.

Boundary spanning behavior was measured using five statements developed and validated by Van Meerkerk and Edelenbos (2014). The measurement instrument asked respondents to indicate the extent to which individuals in their IPU demonstrate boundary spanning behavior in terms of five different boundary spanning activities: good information exchange, building and maintaining sustainable relationships, making effective connections between departments, developing a feeling for what is important in the network, and timely mobilization of the network when necessary. Five-point Likert scales were used, with possible responses ranging from *strongly disagree*(1) to *strongly agree* (5). The total score was calculated by taking the average of the five boundary spanning activity scores. The Cronbach’s alpha coefficient for this scale was .95, indicating excellent reliability.

Task interdependence was measured with three statements taken from the team member questionnaire by van der Vegt (2008). These statements measure the extent to which team members perceive themselves to be interdependent on one another for performing their tasks with answers on the same scale as above for boundary spanning behavior measures. Task interdependence was calculated as the average of the three responses. The Cronbach’s alpha coefficient of the scale was .83, indicating good reliability.

A single-item measure was used to measure time constraints that IPU members experience in their work., Respondents were asked to respond on a 5-point scale, from *never* (1) to *always* (5), as to what degree they experienced time constraints.

Because the collected data are nested (individuals nested within IPUs), a multilevel regression analysis was appropriate (Hox, 1998; Sherry & MacKinnon, 2013). A random-effects analysis was carried out because a Hausman test indicated nonsignificance, and this analysis indicated that the variation in fixed effects across individuals was random and uncorrelated with the models’ independent variables. To test the moderating effects of task interdependence and time constraints, interaction variables were computed based on centered variables. Table 1 provides the descriptive statistics and correlations. This shows a large and significant correlation between profession and education (*r* = .861, *p* < .01), and therefore, no additional control for education was included in the analysis.

## Results

Table 2 shows the results of the multilevel analyses. Several models were used to test the various hypotheses. First, an empty model (Model 0), in which only the variance between teams was included as a predictor of RC, was fitted to the data. This predictor provided insight into the amount of variance in RC scores that was observed between IPUs. The intraclass correlation coefficient (ICC), calculated as the between-cluster variability (= .08) divided by the sum of within-cluster and between-cluster variabilities (= .44), was estimated to be approximately .19. This indicates that about 19% of the variance in RC scores can be explained by differences between teams. On this basis, multilevel analyses were used to predict RC in IPUs.

**Table 2:** Multilevel regression analysis of the relational coordination scores (*n* = 86)

	Model 0 (*b*/*SE*)	Model 1 (*b*/*SE*)	Model 2 (*b*/*SE*)	Model 3 (*b*/*SE*)	Model 4 (*b*/*SE*)	Model 5 (*b*/*SE*)	Model 6 (*b*/*SE*)
Team meetings		.24** (.09)	.22** (.10)	.16 (.10)	.22** (.10)	.23** (.10)	.22** (.10)
Boundary spanning behavior		.06 (.07)	.05 (.07)	.06 (.07)	.05 (.07)	.04 (.07)	.07 (.07)
Task interdependence			.08 (.13)	.12 (.13)	.08 (.13)	.06 (.13)	.07 (.13)
Time constraints			.04 (.07)	.05 (.07)	.04 (.07)	.04 (.07)	.04 (.07)
Team Meetings × Task Interdependence				.32 (.21)			
Team Meetings × Time Constraints					.00 (.10)		
Boundary Spanning Behavior × Task Interdependence						.10 (.15)	
Boundary Spanning Behavior × Time Constraints							.07 (.07)
Constant	3.61** (0.09)	2.53** (0.39)	2.18** (0.59)	2.15** (0.58)	2.19* (0.59)	2.27** (0.60)	2.19** (0.59)
σ^2^_e_ (Level 1, individual)	.36	.57	.58	.56	.52	.56	.54
σ^2^_u0_ (Level 2, IPU)	.08	.28	.27	.26	.24	.26	.25
Log-likelihood	−84.55	−79.93	−79.95	−78.80	−79.95	−79.68	−79.35
Likelihood-ratio test		9.23**	9.20	11.50*	9.20	9.73*	10.39*
*N* Level 1	86	86	86	86	86	86	86
*N* Level 2	26	26	26	26	26	26	26

*Note.* Likelihood-ratio test is used to compare fit of model to Model 0 with only constant. All coefficients from this analysis are derived from the individual level (Level 1).

*p < .10.

**p < .05.

**Figure 1:**
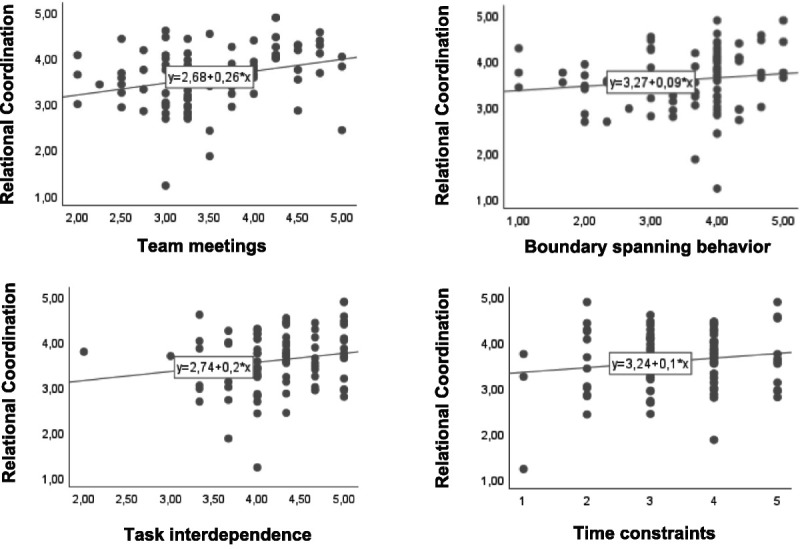
Scatter plots of direct associations between independent variables and the dependent variable.

In Model 1, the number of different professional groups that participated in interprofessional team meetings and the boundary spanning behavior variable were added to the model. The number of different professional groups that participate in interprofessional team meetings has a significant positive association with RC (*b* = .24, *p* < .05). On average, a 1-point increase in the number of professional groups present at interprofessional team meetings is associated with a statistically significant 0.24-point increase in RC score, which is 6.52% of the total variance in RC (0.24/(4.89–1.21) ≈ 6.52). This finding supports Hypothesis 1. The results also show that boundary spanning behavior was not associated with RC (see Figure 1), indicating that Hypothesis 2 is not supported by our study data.

In Model 2, the organizational conditions of task interdependence and time constraints were added to Model 1 to test their direct association with RC. Both task interdependence and time constraints were not associated with RC.

**Figure 2:**
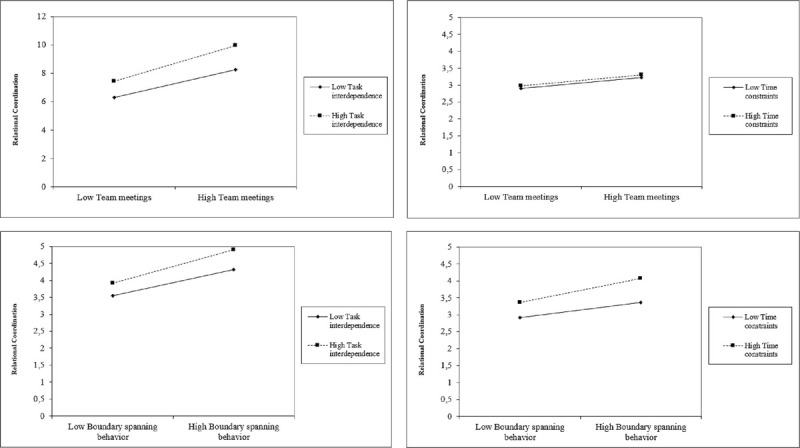
Line graphs of interaction effects. For both boundary spanning behavior and task interdependence, “low” is mean − 1 *SD*, and “high” is mean + 1 *SD*.

Models 3 and 5 include the interaction term of task interdependence and team practices (i.e., the number of professional groups present at team meetings and boundary spanning behavior). These show positive but nonsignificant interaction effects, indicating that H5 and H6 are not supported by our data. Similar nonsignificant results were found in Models 4 and 6 in which the interaction term of time constraints and team practices were included. In Figure 2, we do not observe trends that suggest a potential moderation effect of task interdependency on the association between team meetings and RC. Similarly, no effect of time constraints on the association between boundary spanning behavior and RC can be detected.

To assess the robustness of the study’s findings, we conducted separate analyses for the two dimensions of RC (i.e., communications and relationships). The results from these analyses (see Supplementary Appendix B, http://links.lww.com/HCMR/A130) were consistent with the findings from the main analyses. In addition, the study’s robustness was assessed by comparing the models with and without the demographic variables as controls. Both led to similar results.

## Discussion

This study set out to explore factors that are associated with RC in IPUs by studying (a) the association between team practices, organizational conditions, and RC in IPUs and (b) the moderating effects of organizational conditions. To test the various theory-based hypotheses, a survey was conducted among professionals working in IPUs in two Dutch academic hospitals.

In accordance with RC theory (Gittell, 2006), this study found that the number of professional groups that are present at team meetings is positively associated with the level of RC in IPUs. In other words, having a broader mix of professions at team meetings stimulates the quality of interprofessional communication. These findings suggest that professional groups, by being present at team meetings, have the opportunity to interact with others and thereby communicate and form relationships across professional boundaries, which increases RC (Galbraith, 1974; Hartgerink et al., 2014; Lamb et al., 2011). On a note of caution, the cross-sectional design of the survey means that a reverse relationship between RC and team meetings cannot be ruled out; it is plausible that professionals who achieve high-quality communication or strong relationships are more inclined to be present at team meetings. It is also possible that team meetings and RC are mutually reinforcing. Future research should focus on examining the underlying mechanisms that contribute to the relationship between the number of professional groups present at IPU team meetings and RC.

Contrary to our expectations, our findings do not provide support for a positive relationship between boundary spanning behavior and RC. The lack of a statistical significant result may be attributed to the limited statistical power of this study. To further investigate the potential relationship between boundary spanning behavior and RC in IPUs, future research should investigate this relationship in a larger sample size. This discrepancy between the theoretical framework and the results of this study might also be due to how we operationalized boundary spanning. In this study, boundary spanning behavior was operationalized as boundary spanning behavior as expressed by IPU members. In some studies, boundary spanning has been operationalized as a well-defined and officially recognized boundary spanning role. Some studies adopting this approach have found that establishing such a boundary spanning role resulted in higher levels of RC (Di Capua et al., 2017; Skakon, 2014). Consequently, it would be beneficial to examine the association between designated boundary spanning roles, boundary spanning behavior, and RC in IPUs.

The findings in this study suggest that experiencing task interdependence and time constraints do not significantly influence RC among IPU members. Although our findings suggest that task interdependence is indeed high among IPU members, our findings also indicate that there is little variation in levels of task interdependence across IPU members (*M* = 4.22, *SD* = 0.57), which could explain the failure to identify a relationship. Another plausible explanation for these results is that, in IPUs, everybody’s work is highly task interdependent and delivered under time constraints and that, under these circumstances, IPU members strive to deliver the full cycle of care for the patient, regardless of their sense of task interdependence or time constraints. This suggests that the influence of task interdependence and time constraints on RC might be moderated by the adaptation of IPU members to these conditions.

Based on the findings in this study, no patterns were observed regarding the influence of the conditional factors (i.e., task interdependency and time constraints) on the association between the team practices (i.e., team meetings and boundary spanning behavior) and RC in IPUs. To further explore the relationships between the team practices, conditional factors, and RC in IPUs, our research could be extended to a larger number of IPUs. In addition, extending the investigation into the antecedents of RC could involve examining their impact on the performance of IPUs. In this way, organizations could better understand how to intervene to increase IPU performance by improving RC.

Our study on RC in IPUs not only adds to earlier studies on RC, it also contributes to current insights into VBHC in several ways. This study has shown that mapping relational dynamics can generate insight into the actual practices of interprofessional collaboration in IPUs. As such, understanding how professionals work in IPUs can identify factors that determine the success or failure of VBHC at the microlevel of professionals and IPUs. This is important as this is currently lacking at this level of analysis, as is guidance for managing social processes on the group level in VBHC theory and practice (van Staalduinen et al., 2022). As such, RC could be used as a framework to enhance interprofessional collaboration within IPUs. To this end, future research is needed that explores and tests interventions that promote RC among IPU members.

A few limitations of this study need to be acknowledged. First, the findings are based on a small sample, and this increases the likelihood of insufficient statistical power. The small sample size and accompanying low statistical power might have played a role in finding nonsignificant associations within the current study, and in general, the results may be less reliable. Despite our efforts to achieve a large sample size, this remained small, in part, because eligible participants failed to complete the survey either because they did not consider themselves part of the IPU or felt they did not collaborate enough with other professional groups. Consequently, the current study should be viewed as an exploratory study on which future studies could build.

Second, although we controlled for the nested structure, we were unable to distinguish between group level constructs because of the sample size. Therefore, some variables that are in fact team-level constructs in this study are analyzed on the individual level. The organizational literature argues that individuals are tied to their teams and that individual-level characteristics influence the group-level characteristics, and vice versa (Rousseau, 1985). Therefore, not being able to distinguish between constructs at the group level may have influenced the findings in this study. Given that our results are promising (see Figures 1 and 2) and that interprofessional collaboration in IPUs is under researched, future studies among larger numbers of IPUs could usefully test and further specify hypotheses on the antecedents and effects of RC.

## Practice Implications

First, it is important for managers to explore ways to improve collaboration among professionals in interprofessional collaborations, such as IPUs. The varying levels of RC observed in this study indicate that there is still room for managers to work on the quality of collaboration. A first step in this process could be to involve discussing levels of RC among members of an IPU.

Second, our findings highlight that the presence of diverse professional groups in IPU meetings positively influences the interaction among IPU members and thus RC. Therefore, we recommend that IPU members prioritize attending these meetings whereas health care managers should actively encourage and motivate IPU members to participate in them, emphasizing the importance of collectively discussing IPU practices.

Third, in addition to fostering employee engagement, health care managers can provide IPU members opportunities to participate in IPU meetings. They can facilitate this by offering structural support, such as organizing dedicated platforms for IPU meetings and appointing a responsible person to plan and coordinate these meetings.

## Acknowledgments

We thank the Erasmus University Medical Center and in particular Prof. Dr. Arie Franx for his assistance in collecting data for this study.

## Footnotes

1 Earlier research describes an IPU as an ideal type of organizational unit in that it matches VBHC theory concerning major organizational shifts (Porter & Lee, 2021; Steinmann et al., 2022). The IPUs in this study are similar to IPUs as described in VBHC theory but do not have the suggested financial accountability.
